# *N*′-[(5-Chloro-1*H*-indol-3-yl)methyl­ene]-3,4,5-trihydroxy­benzohydrazide

**DOI:** 10.1107/S160053680804258X

**Published:** 2008-12-20

**Authors:** Hamid Khaledi, Hapipah Mohd Ali, Seik Weng Ng

**Affiliations:** a Department of Chemistry, University of Malaya, 50603 Kuala Lumpur, Malaysia

## Abstract

The two aromatic parts of the title compound, C_16_H_13_ClN_3_O_4_, are connected through a conjugated –CH=N–NH–C(O)– fragment, giving an almost planar mol­ecule (r.m.s. deviation 0.08 Å). In the crystal structure, adjacent mol­ecules are linked by N—H⋯O and O—H⋯O hydrogen bonds into a three-dimensional network.

## Related literature

For the isostructural C_16_H_13_BrN_3_O_4_ analog, see: Khaledi *et al.* (2008 ▶).
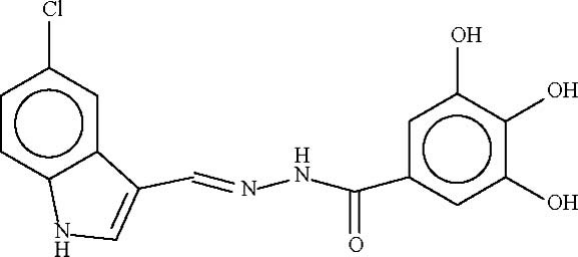


## Experimental

### 

#### Crystal data

C_16_H_12_ClN_3_O_4_
*M* *_r_* = 345.74
Monoclinic, 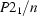

*a* = 9.6481 (2) Å
*b* = 15.1408 (3) Å
*c* = 10.2206 (2) Å
β = 98.232 (1)°
*V* = 1477.64 (5) Å^3^
*Z* = 4
Mo *K*α radiation
μ = 0.29 mm^−1^
*T* = 100 (2) K
0.32 × 0.22 × 0.12 mm

#### Data collection

Bruker SMART APEX diffractometer
Absorption correction: multi-scan (*SADABS*; Sheldrick, 1996 ▶) *T*_min_ = 0.914, *T*_max_ = 0.966
10185 measured reflections
3389 independent reflections
2907 reflections with *I* > 2σ(*I*)
*R*_int_ = 0.025

#### Refinement

*R*[*F* ^2^ > 2σ(*F* ^2^)] = 0.034
*wR*(*F* ^2^) = 0.093
*S* = 1.06
3389 reflections
220 parameters
H-atom parameters constrained
Δρ_max_ = 0.34 e Å^−3^
Δρ_min_ = −0.33 e Å^−3^

### 

Data collection: *APEX2* (Bruker, 2007 ▶); cell refinement: *SAINT* (Bruker, 2007 ▶); data reduction: *SAINT*; program(s) used to solve structure: *SHELXS97* (Sheldrick, 2008 ▶); program(s) used to refine structure: *SHELXL97* (Sheldrick, 2008 ▶); molecular graphics: *X-SEED* (Barbour, 2001 ▶); software used to prepare material for publication: *publCIF* (Westrip, 2009 ▶).

## Supplementary Material

Crystal structure: contains datablocks global, I. DOI: 10.1107/S160053680804258X/xu2471sup1.cif

Structure factors: contains datablocks I. DOI: 10.1107/S160053680804258X/xu2471Isup2.hkl

Additional supplementary materials:  crystallographic information; 3D view; checkCIF report

## Figures and Tables

**Table 1:** Hydrogen-bond geometry (Å, °)

*D*—H⋯*A*	*D*—H	H⋯*A*	*D*⋯*A*	*D*—H⋯*A*
O2—H2o⋯O3	0.84	2.20	2.6726 (14)	116
O3—H3o⋯O1^i^	0.84	1.74	2.5818 (14)	175
O4—H4o⋯N2^i^	0.84	2.01	2.7668 (15)	149
N1—H1n⋯O2^ii^	0.88	2.23	3.0875 (16)	163
N3—H3n⋯O4^iii^	0.88	2.15	2.9518 (15)	152

Symmetry codes: (i) 
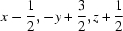
; (ii) 
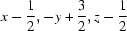
; (iii) 
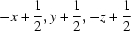
.

**Fig. 1.**
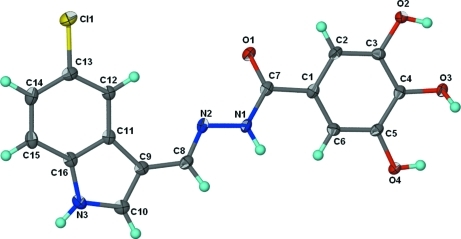
Thermal ellipsoid plot (Barbour, 2001) of C16H12ClN3O4 at the 70% probability level. Hydrogen atoms are drawn as spheres of arbitrary radius.

## supplementary crystallographic information

### Experimental

5-Chloroindole-3-carbaldehyde (0.27 g, 1.5 mmol) and 3,4,5-trihydroxybenzoylhydrazine (0.27 g, 1.5 mmol) were heated in ethanol (20 ml) for 3 h. About 1 ml of acetic acid also added. The solid that separated out was collected, washed with water and then recrystallized from DMSO.

### Refinement

Hydrogen atoms were placed at calculated positions (C–H 0.95, N–H 0.88, O–H 0.84 Å) and were treated as riding on their parent carbon atoms, with *U*(H) set to 1.2–1.5 times *U*_eq_(*C*,*N*,*O*). The hydroxy groups were rotated to fit the electron density.

### Crystal data

**Table:**

C_16_H_12_ClN_3_O_4_	*F*(000) = 712
*M**_r_* = 345.74	*D*_x_ = 1.554 Mg m^−^^3^
Monoclinic, *P*2_1_/*n*	Mo *K*α radiation, λ = 0.71073 Å
Hall symbol: -P 2yn	Cell parameters from 4113 reflections
*a* = 9.6481 (2) Å	θ = 2.4–28.2°
*b* = 15.1408 (3) Å	µ = 0.29 mm^−^^1^
*c* = 10.2206 (2) Å	*T* = 100 K
β = 98.232 (1)°	Prism, orange
*V* = 1477.64 (5) Å^3^	0.32 × 0.22 × 0.12 mm
*Z* = 4	

### Data collection

**Table:**

Bruker SMART APEX diffractometer	3389 independent reflections
Radiation source: fine-focus sealed tube	2907 reflections with *I* > 2σ(*I*)
graphite	*R*_int_ = 0.025
ω scans	θ_max_ = 27.5°, θ_min_ = 2.4°
Absorption correction: multi-scan (*SADABS*; Sheldrick, 1996)	*h* = −11→12
*T*_min_ = 0.914, *T*_max_ = 0.966	*k* = −19→19
10185 measured reflections	*l* = −13→13

### Refinement

**Table:**

Refinement on *F*^2^	Primary atom site location: structure-invariant direct methods
Least-squares matrix: full	Secondary atom site location: difference Fourier map
*R*[*F*^2^ > 2σ(*F*^2^)] = 0.034	Hydrogen site location: inferred from neighbouring sites
*wR*(*F*^2^) = 0.093	H-atom parameters constrained
*S* = 1.06	*w* = 1/[σ^2^(*F*_o_^2^) + (0.0452*P*)^2^ + 0.6635*P*] where *P* = (*F*_o_^2^ + 2*F*_c_^2^)/3
3389 reflections	(Δ/σ)_max_ = 0.001
220 parameters	Δρ_max_ = 0.34 e Å^−^^3^
0 restraints	Δρ_min_ = −0.33 e Å^−^^3^

### Fractional atomic coordinates and isotropic or equivalent isotropic displacement parameters (Å^2^)

**Table:**

	*x*	*y*	*z*	*U*_iso_*/*U*_eq_	
Cl1	0.99034 (4)	1.07558 (3)	0.24671 (4)	0.02606 (12)	
O1	0.68592 (10)	0.82901 (7)	0.56884 (10)	0.0165 (2)	
O2	0.64086 (10)	0.62519 (7)	0.94667 (10)	0.0172 (2)	
H2O	0.5962	0.5961	0.9968	0.026*	
O3	0.36728 (11)	0.59550 (6)	0.94304 (9)	0.0140 (2)	
H3O	0.3047	0.6195	0.9801	0.021*	
O4	0.16964 (10)	0.67781 (7)	0.73963 (10)	0.0149 (2)	
H4O	0.1521	0.6414	0.7971	0.022*	
N1	0.46397 (12)	0.86816 (8)	0.48430 (11)	0.0138 (2)	
H1N	0.3740	0.8638	0.4895	0.017*	
N2	0.51003 (13)	0.92205 (7)	0.38882 (11)	0.0137 (2)	
N3	0.39990 (13)	1.10793 (8)	0.03144 (12)	0.0162 (3)	
H3N	0.3544	1.1361	−0.0369	0.019*	
C1	0.50119 (15)	0.76183 (9)	0.66318 (13)	0.0130 (3)	
C2	0.59758 (15)	0.72084 (9)	0.75913 (13)	0.0141 (3)	
H2	0.6952	0.7305	0.7616	0.017*	
C3	0.54846 (15)	0.66576 (9)	0.85061 (13)	0.0131 (3)	
C4	0.40551 (15)	0.65121 (9)	0.84928 (13)	0.0123 (3)	
C5	0.31087 (14)	0.69082 (9)	0.75114 (13)	0.0125 (3)	
C6	0.35827 (15)	0.74599 (9)	0.65851 (13)	0.0133 (3)	
H6	0.2932	0.7730	0.5919	0.016*	
C7	0.55811 (15)	0.82260 (9)	0.56894 (13)	0.0131 (3)	
C8	0.41317 (15)	0.96681 (9)	0.31823 (13)	0.0142 (3)	
H8	0.3195	0.9615	0.3358	0.017*	
C9	0.44207 (15)	1.02444 (9)	0.21398 (13)	0.0142 (3)	
C10	0.33977 (16)	1.05950 (9)	0.11972 (14)	0.0162 (3)	
H10	0.2419	1.0509	0.1172	0.019*	
C11	0.57451 (15)	1.05429 (9)	0.18016 (13)	0.0133 (3)	
C12	0.71420 (15)	1.04422 (9)	0.23767 (13)	0.0147 (3)	
H12	0.7386	1.0109	0.3164	0.018*	
C13	0.81494 (16)	1.08455 (10)	0.17549 (14)	0.0169 (3)	
C14	0.78385 (16)	1.13505 (10)	0.06023 (14)	0.0187 (3)	
H14	0.8574	1.1609	0.0206	0.022*	
C15	0.64682 (16)	1.14724 (9)	0.00445 (14)	0.0172 (3)	
H15	0.6235	1.1823	−0.0727	0.021*	
C16	0.54321 (16)	1.10616 (9)	0.06539 (13)	0.0146 (3)	

### Atomic displacement parameters (Å^2^)

**Table:**

	*U*^11^	*U*^22^	*U*^33^	*U*^12^	*U*^13^	*U*^23^
Cl1	0.01421 (19)	0.0351 (2)	0.0286 (2)	−0.00330 (15)	0.00218 (15)	0.00450 (16)
O1	0.0125 (5)	0.0194 (5)	0.0187 (5)	−0.0004 (4)	0.0054 (4)	0.0023 (4)
O2	0.0113 (5)	0.0246 (6)	0.0156 (5)	0.0001 (4)	0.0013 (4)	0.0057 (4)
O3	0.0132 (5)	0.0167 (5)	0.0131 (5)	0.0012 (4)	0.0055 (4)	0.0018 (4)
O4	0.0101 (5)	0.0191 (5)	0.0155 (5)	−0.0022 (4)	0.0017 (4)	0.0035 (4)
N1	0.0124 (6)	0.0149 (6)	0.0152 (6)	−0.0011 (5)	0.0059 (4)	0.0021 (4)
N2	0.0159 (6)	0.0135 (6)	0.0129 (5)	−0.0014 (5)	0.0060 (4)	−0.0005 (4)
N3	0.0185 (6)	0.0160 (6)	0.0135 (5)	0.0028 (5)	0.0003 (5)	0.0009 (4)
C1	0.0145 (7)	0.0119 (6)	0.0134 (6)	−0.0007 (5)	0.0050 (5)	−0.0029 (5)
C2	0.0110 (7)	0.0166 (7)	0.0154 (6)	−0.0016 (5)	0.0039 (5)	−0.0022 (5)
C3	0.0122 (7)	0.0143 (6)	0.0125 (6)	0.0024 (5)	0.0009 (5)	−0.0020 (5)
C4	0.0140 (7)	0.0126 (6)	0.0110 (6)	−0.0013 (5)	0.0038 (5)	−0.0024 (5)
C5	0.0109 (6)	0.0137 (6)	0.0135 (6)	−0.0009 (5)	0.0034 (5)	−0.0038 (5)
C6	0.0134 (7)	0.0137 (6)	0.0125 (6)	0.0010 (5)	0.0014 (5)	−0.0007 (5)
C7	0.0151 (7)	0.0121 (6)	0.0131 (6)	−0.0004 (5)	0.0049 (5)	−0.0040 (5)
C8	0.0132 (7)	0.0149 (6)	0.0153 (6)	−0.0012 (5)	0.0046 (5)	−0.0039 (5)
C9	0.0153 (7)	0.0136 (6)	0.0139 (6)	0.0004 (5)	0.0029 (5)	−0.0029 (5)
C10	0.0164 (7)	0.0155 (7)	0.0167 (7)	0.0003 (6)	0.0020 (5)	−0.0032 (5)
C11	0.0173 (7)	0.0102 (6)	0.0130 (6)	−0.0005 (5)	0.0041 (5)	−0.0028 (5)
C12	0.0167 (7)	0.0134 (7)	0.0139 (6)	−0.0003 (5)	0.0024 (5)	−0.0005 (5)
C13	0.0143 (7)	0.0173 (7)	0.0193 (7)	−0.0007 (6)	0.0024 (6)	−0.0022 (5)
C14	0.0224 (8)	0.0159 (7)	0.0192 (7)	−0.0037 (6)	0.0082 (6)	−0.0005 (5)
C15	0.0255 (8)	0.0133 (6)	0.0137 (6)	−0.0004 (6)	0.0058 (6)	−0.0002 (5)
C16	0.0191 (7)	0.0122 (6)	0.0125 (6)	0.0003 (5)	0.0022 (5)	−0.0028 (5)

### Geometric parameters (Å, °)

**Table:**

Cl1—C13	1.7491 (15)	C2—H2	0.9500
O1—C7	1.2371 (17)	C3—C4	1.3948 (19)
O2—C3	1.3731 (16)	C4—C5	1.3919 (19)
O2—H2O	0.8400	C5—C6	1.3883 (19)
O3—C4	1.3664 (16)	C6—H6	0.9500
O3—H3O	0.8400	C8—C9	1.4350 (19)
O4—C5	1.3650 (16)	C8—H8	0.9500
O4—H4O	0.8400	C9—C10	1.383 (2)
N1—C7	1.3506 (18)	C9—C11	1.443 (2)
N1—N2	1.3927 (16)	C10—H10	0.9500
N1—H1N	0.8800	C11—C12	1.399 (2)
N2—C8	1.2884 (19)	C11—C16	1.4080 (19)
N3—C10	1.3558 (19)	C12—C13	1.378 (2)
N3—C16	1.3768 (19)	C12—H12	0.9500
N3—H3N	0.8800	C13—C14	1.400 (2)
C1—C6	1.3936 (19)	C14—C15	1.375 (2)
C1—C2	1.397 (2)	C14—H14	0.9500
C1—C7	1.4925 (19)	C15—C16	1.397 (2)
C2—C3	1.3860 (19)	C15—H15	0.9500
			
C3—O2—H2O	109.5	O1—C7—C1	120.52 (13)
C4—O3—H3O	109.5	N1—C7—C1	116.90 (12)
C5—O4—H4O	109.5	N2—C8—C9	122.22 (13)
C7—N1—N2	119.69 (12)	N2—C8—H8	118.9
C7—N1—H1N	120.2	C9—C8—H8	118.9
N2—N1—H1N	120.2	C10—C9—C8	123.75 (14)
C8—N2—N1	114.96 (12)	C10—C9—C11	106.32 (12)
C10—N3—C16	109.38 (12)	C8—C9—C11	129.87 (13)
C10—N3—H3N	125.3	N3—C10—C9	109.91 (13)
C16—N3—H3N	125.3	N3—C10—H10	125.0
C6—C1—C2	120.24 (13)	C9—C10—H10	125.0
C6—C1—C7	122.53 (13)	C12—C11—C16	119.31 (13)
C2—C1—C7	117.23 (13)	C12—C11—C9	134.24 (13)
C3—C2—C1	118.90 (13)	C16—C11—C9	106.41 (13)
C3—C2—H2	120.5	C13—C12—C11	117.29 (13)
C1—C2—H2	120.5	C13—C12—H12	121.4
O2—C3—C2	120.11 (12)	C11—C12—H12	121.4
O2—C3—C4	118.44 (12)	C12—C13—C14	123.35 (14)
C2—C3—C4	121.44 (13)	C12—C13—Cl1	118.55 (11)
O3—C4—C5	123.80 (13)	C14—C13—Cl1	118.07 (11)
O3—C4—C3	117.16 (12)	C15—C14—C13	119.96 (14)
C5—C4—C3	118.99 (12)	C15—C14—H14	120.0
O4—C5—C6	116.76 (12)	C13—C14—H14	120.0
O4—C5—C4	122.94 (12)	C14—C15—C16	117.53 (13)
C6—C5—C4	120.30 (13)	C14—C15—H15	121.2
C5—C6—C1	120.07 (13)	C16—C15—H15	121.2
C5—C6—H6	120.0	N3—C16—C15	129.48 (13)
C1—C6—H6	120.0	N3—C16—C11	107.98 (12)
O1—C7—N1	122.58 (13)	C15—C16—C11	122.54 (14)
			
C7—N1—N2—C8	−175.96 (12)	N2—C8—C9—C10	166.52 (13)
C6—C1—C2—C3	−1.2 (2)	N2—C8—C9—C11	−10.3 (2)
C7—C1—C2—C3	178.31 (12)	C16—N3—C10—C9	−0.12 (16)
C1—C2—C3—O2	−179.47 (12)	C8—C9—C10—N3	−177.25 (12)
C1—C2—C3—C4	−0.5 (2)	C11—C9—C10—N3	0.18 (15)
O2—C3—C4—O3	−1.36 (18)	C10—C9—C11—C12	177.59 (15)
C2—C3—C4—O3	179.69 (12)	C8—C9—C11—C12	−5.2 (3)
O2—C3—C4—C5	−178.98 (12)	C10—C9—C11—C16	−0.17 (15)
C2—C3—C4—C5	2.1 (2)	C8—C9—C11—C16	177.04 (14)
O3—C4—C5—O4	0.2 (2)	C16—C11—C12—C13	−1.65 (19)
C3—C4—C5—O4	177.70 (12)	C9—C11—C12—C13	−179.19 (14)
O3—C4—C5—C6	−179.32 (12)	C11—C12—C13—C14	0.7 (2)
C3—C4—C5—C6	−1.9 (2)	C11—C12—C13—Cl1	178.50 (10)
O4—C5—C6—C1	−179.44 (12)	C12—C13—C14—C15	0.8 (2)
C4—C5—C6—C1	0.2 (2)	Cl1—C13—C14—C15	−176.97 (11)
C2—C1—C6—C5	1.4 (2)	C13—C14—C15—C16	−1.3 (2)
C7—C1—C6—C5	−178.09 (12)	C10—N3—C16—C15	−179.12 (14)
N2—N1—C7—O1	2.73 (19)	C10—N3—C16—C11	0.00 (15)
N2—N1—C7—C1	−176.29 (11)	C14—C15—C16—N3	179.40 (14)
C6—C1—C7—O1	−173.65 (13)	C14—C15—C16—C11	0.4 (2)
C2—C1—C7—O1	6.83 (19)	C12—C11—C16—N3	−178.05 (12)
C6—C1—C7—N1	5.39 (19)	C9—C11—C16—N3	0.11 (15)
C2—C1—C7—N1	−174.13 (12)	C12—C11—C16—C15	1.1 (2)
N1—N2—C8—C9	−178.92 (12)	C9—C11—C16—C15	179.30 (13)

### Hydrogen-bond geometry (Å, °)

**Table:**

*D*—H···*A*	*D*—H	H···*A*	*D*···*A*	*D*—H···*A*
O2—H2o···O3	0.84	2.20	2.6726 (14)	116
O3—H3o···O1^i^	0.84	1.74	2.5818 (14)	175
O4—H4o···N2^i^	0.84	2.01	2.7668 (15)	149
N1—H1n···O2^ii^	0.88	2.23	3.0875 (16)	163
N3—H3n···O4^iii^	0.88	2.15	2.9518 (15)	152

Symmetry codes: (i) *x*−1/2, −*y*+3/2, *z*+1/2; (ii) *x*−1/2, −*y*+3/2, *z*−1/2; (iii) −*x*+1/2, *y*+1/2, −*z*+1/2.
